# A Bayesian Approach to the Analysis of Local Average Treatment Effect for Missing and Non-normal Data in Causal Modeling: A Tutorial With the ALMOND Package in R

**DOI:** 10.3389/fpsyg.2020.00169

**Published:** 2020-02-18

**Authors:** Dingjing Shi, Xin Tong, M. Joseph Meyer

**Affiliations:** ^1^Department of Psychology, University of Oklahoma, Norman, OK, United States; ^2^Department of Psychology, University of Virginia, Charlottesville, VA, United States

**Keywords:** instrumental variables (IV), bayesian method, robust method, missing data, selection bias, R package, causal modeling, local average treatment effect

## Abstract

One practical challenge in observational studies and quasi-experimental designs is selection bias. The issue of selection bias becomes more concerning when data are non-normal and contain missing values. Recently, a Bayesian robust two-stage causal modeling with instrumental variables was developed and has the advantages of addressing selection bias and handle non-normal data and missing data simultaneously in one model. The method provides reliable parameter and standard error estimates when missing data and outliers exist. The modeling technique can be widely applied to empirical studies particularly in social, psychological and behavioral areas where any of the three issues (e.g., selection bias, data with outliers and missing data) is commonly seen. To implement this method, we developed an R package named ALMOND (**A**nalysis of **L**ATE (Local Average Treatment Effect) for **M**issing **O**r/and **N**onnormal **D**ata). Package users have the flexibility to directly apply the Bayesian robust two-stage causal models or write their own Bayesian models from scratch within the package. To facilitate the application of the Bayesian robust two-stage causal modeling technique, we provide a tutorial for the ALMOND package in this article, and illustrate the application with two examples from empirical research.

In observational studies or quasi-experimental designs, when there are omitted variables or confounder effects, the regression coefficient estimates in the causal model are biased (e.g., Angrist and Keueger, 1991; Angrist and Pischke, 2014), which is a source of selection bias. One strategy to address the selection bias is to introduce instrumental variables (InsV) into the analytic model (Angrist and Pischke, 2008). In particular, in the presence of confounder effects, the changes in the treatment are associated not only with changes in the outcome but also with changes in the error. The two associations/variations in the treatment variable eventually lead to an inconsistent estimate of the treatment effect. Incorporating InsVs in the analytic model is a frequently used and effective way to separate the two variations in the causal treatment: InsVs separate the variations of the treatment effects that are associated with the causal outcome from the variations in the treatment that are associated with the model residuals. In other words, InsVs are factors that cause some variations in the treatment variable but have no associations with the model residuals. When InsVs are incorporated in the analytical model, changes in InsVs are associated only with the changes in the treatment but not with the error. The only association between the InsVs and the outcome variables is via the indirect route of the treatment variable (Cameron and Trivedi, 2005).

InsVs play an instrumental role in the causal chain between the treatment and the outcome variable. At least two properties qualify variables as InsVs. First, the instrument needs to be related to the treatment so that the associated part between the instrument and the treatment explains variations of the outcome that contributes to the biasedness of the coefficient estimates. In addition, the instrument has to only affect the outcome through the treatment, but via no other route. In this way, the causal chain is established and only a partial causal effect of the treatment that has not been contaminated by the confounder effects will be used to estimate the treatment effect to the outcome (e.g., Baiocchi et al., 2014). This partial treatment effect is often called the Local Average Treatment Effect (LATE) (Angrist et al., 1996) and is often the center of research interest in the causal model.

There have been methodological advances to incorporate InsVs (hereafter called *InsV methods*), such as two-stage least squares (2SLS) estimator (Hägglund, 1982; Jöreskog, 1983; Angrist and Imbens, 1995; Bound et al., 1995; Acemoglu and Angrist, 1999; Breierova and Duflo, 2004; Hudson and Sessions, 2011; Angrist and Pischke, 2014) and maximum likelihood estimator (Mariano and McDonald, 1979; Bollen et al., 2007; Maydeu-Olivares et al., 2019), as well as applications of these methods in empirical studies (e.g., Currie and Yelowitz, 2000; Revelli, 2002; Miguel et al., 2004; Rassen et al., 2009). Crespo-Tenorio and Montgomery (2013) proposed a Bayesian method to incorporate InsVs to improve estimation, particularly with weak instruments and small samples. There have also been Bayesian approaches to study InsV methods with various types of outcome variables (Burgess and Thompson, 2012; Li and Lu, 2015). However, as far as we are aware, the number of software packages available to help implement the InsV methods is limited. A common choice for researchers using InsV methods is the InsV feature in Stata (StataCorp, 2017). An equivalent option is the AER package in R (Kleiber et al., 2018). The sem package in R has a side ability to incorporate InsVs in the model by calling the AER package. The lavaan package (Rosseel, 2012) allows users to incorporate InsVs in the structural equation modeling framework. All these software programs or packages adopt the InsV methods from the frequentist perspective and seldom deal with contaminated (e.g., non-normal or missing) data.

Recently, a Bayesian robust two-stage causal model with InsVs was proposed to account for selection bias and simultaneously handle the non-normal and/or missing data in one model and provide reliable parameter estimates (Shi and Tong, accepted). In the presence of omitted confounders, the regression coefficient estimates will be biased. Regression coefficient estimates can be biased when there are non-normal or missing data. In the simultaneous presence of omitted confounders and non-normal or missing data, the bias in regression coefficients can get worse, which may eventually have an impact on selection bias. In the proposed method, InsVs were carefully chosen to disentangle the partial treatment effect of research interests or the LATE. By estimating the LATE, the generalizability of the study (i.e., external validity) was traded for an improved consistent estimate (i.e., internal validity) of the treatment effects. Furthermore, robust methods based on Student's *t* distributions were introduced to model data containing outliers. Multiple imputation techniques were used to handle ignorable missing data. For non-ignorable missing data, an added-on selection model was applied so that the missingness in the outcome variable was explained via a link function. Bayesian methods were used for the estimation. The conventional 2SLS model was also applied to the same data and it was found that Bayesian methods performed as equally well as the 2SLS model under ideal data conditions (i.e., normally distributed and complete). Monte Carlo simulation studies showed that the proposed method outperformed conventional frequentist approaches under contaminated data conditions. Particularly, the robust models outperformed the corresponding normal-based models for data containing outliers. When data are ignorable missing, the two-stage Bayesian approach can estimate the LATE well. For the non-ignorable missing data, an added-on selection model performed much better than its non-selection counterpart when estimating the LATE.

This new modeling technique can be applied in a more widespread manner to empirical studies than traditional InsV methods. In addition to addressing selection bias, the model simultaneously handles data containing outliers and missing values, which are commonly seen in social and behavioral research. That being said, this new model may not be easily applicable as it incorporates several advanced techniques in one model. On top of that, Bayesian methods are used, which may push away potential users who are more comfortable with frequentist approaches. In this endeavor, we recently developed an R package to facilitate the application of the Bayesian robust two-stage causal model with InsVs. The package is used for the **A**nalysis of the **L**ATE (Local Average Treatment Effect) with **M**issing **O**r\and **N**onnormal **D**ata, named as ALMOND. In summary, the package adopts a Bayesian approach to incorporate InsVs using a robust two-stage causal modeling framework to estimate the LATE and can simultaneously address the issues of non-normal and missing data. Bayesian estimation methods have many advantages, e.g., handling both ignorable and non-ignorable missing data relatively easily, being more precise than frequentist estimations when the sample size is small, and providing reliable standard error estimates (Shi and Tong, 2018), etc. The ALMOND package fills the methodology gap by adding the Bayesian approach to existing software or packages that use InsVs to address the selection bias. The package is also more powerful than existing software programs as it can provide reliable standard errors for non-normal data containing missing values. Current Bayesian software or packages either have users write their own Bayesian models (e.g., Plummer et al., 2003; Lunn et al., 2009) from scratch or automatically fit certain Bayesian models for the users (e.g., Merkle and Rosseel, 2018). Either approach has its own flaw as writing one's Bayesian model may make the application less accessible to a general audience, whereas fitting a default Bayesian model will be less flexible to users who are more advanced with Bayesian methods. The ALMOND package has the flexibility of doing both options, which can well satisfy the needs of a broader audience from applied researchers to Bayesian methodologists. In this article, we provide a tutorial for this package.

The article is organized as follows. In the subsequent section, the robust two-stage causal modeling with missing data is briefly introduced. Then a detailed decomposition of the package is discussed. The following section illustrates the application of the Bayesian two-stage causal models to two empirical examples using the ALMOND package. The article concludes with a summary and discussion of the package.

## 1. Bayesian Robust Two-Stage Causal Modeling With Missing Data

A two-stage modeling procedure is used to incorporate InsVs. Let *X*_*i*_ and *Y*_*i*_ be the treatment and the outcome for individual *i* (*i* = 1, …, *N*), respectively, and ${\text{Z}}_{i}={\left(Z_{i1},\ldots ,Z_{iJ}\right)}^{\prime }$ be a vector of InsVs. *N* is the sample size and *J* is the total number of instrumental variables. In the first stage of the model, the InsVs **Z** are used to predict the treatment *X*. In other words, the portion of variations in the treatment *X* is identified and estimated by the InsVs **Z**; then, the second stage relies on the estimated exogenous proportion of treatment variations in the form of the predicted treatment values to estimate the treatment effect on the outcome *Y*.

A Bayesian robust two-stage causal modeling approach has been recently proposed (Shi and Tong, accepted) and the framework has two general types of linear models, which accommodate the continuous and categorical (i.e., dichotomous) treatment variables, respectively. For continuous treatment variables, a mathematical form of the general two-stage causal model is represented as:

(1) $$\begin{array}{l} X_{i}=\pi_{10}+\pi_{11}{\text{Z}}_{i}+e_{1i}, \end{array}$$

(2) $$\begin{array}{l} Y_{i}=\pi_{20}+\pi_{21}{\hat{X}}_{i}+e_{2i}. \end{array}$$

In the first stage, π_10_ and $\pi_{11}={\left(\pi_{11},\ldots ,\pi_{1J}\right)}^{\prime }$ are the intercept and regression coefficients, respectively, for the linear model where the treatment *X* is regressed on the InsVs **Z**; in the second stage, π_20_ and π_21_ are the intercept and slope, respectively, for the linear model where the outcome *Y* is regressed on the predicted treatment values of $\hat{X}$. **π**_11_ is the causal effect of the InsVs **Z** on the treatment *X*; and π_21_ is the treatment effect on the outcome *Y* for a subset of participants whose treatment effect has been extracted and explained by the InsVs **Z**. Traditionally, the residuals *e*_1*i*_ and *e*_2*i*_, are assumed to be normally distributed as $e_{1i}\sim N\left(0,\sigma_{e1}^{2}\right)$ and $e_{2i}\sim N\left(0,\sigma_{e2}^{2}\right)$, where *e*_1*i*_ and *e*_2*i*_ are residuals from the general two-stage causal model.

When the treatment variable is dichotomous, a generalized two-stage causal model can be expressed as:

(3) $$\begin{array}{l} X_{i}\sim Bernoulli\left(q_{i}\right), \end{array}$$

(4) $$\begin{array}{l} logit\left(q_{i}\right)=\pi_{10}+\pi_{11}{\text{Z}}_{i}, \end{array}$$

(5) $$\begin{array}{l} Y_{i}=\pi_{20}+\pi_{21}{\hat{X}}_{i}+e_{i}. \end{array}$$

where π_10_ and $\pi_{11}={\left(\pi_{11},\ldots ,\pi_{1J}\right)}^{\prime }$ are the intercept and regression coefficients, respectively, for a logistic regression model where the logia of the probability that the participant is in the treatment group is regressed on the InsVs **Z**. The treatment variable *X*_*i*_ follows a Bernoulli distribution with *q*_*i*_ as the conditional probability that *X*_*i*_ = 1 (participant being in the treatment group), and 1 − *q*_*i*_ as the probability that *X*_*i*_ = 0 (participant being in the control group). In traditional generalized two-stage causal models, the residual *e*_*i*_ at the second stage is assumed to be normally distributed as $e_{i}\sim N\left(0,\sigma^{2}\right)$, where *e*_*i*_ is the residual from the generalized two-stage causal model. Note that covariates can also be added and controlled for in the second stage.

Depending on whether the outcome data are normally distributed or not, the traditional two-stage causal models can be extended to robust models by assuming the error term in stage two follows Student's *t* distributions. Therefore, when the treatment variable is continuous, there are two types of models: the cont-normal-based model (M1) where errors are normally distributed at the second stage ($e_{2i}\sim N\left(0,\sigma_{e2}^{2}\right)$) and the cont-robust model (M2) with *t* distributed errors at the second stage ($e_{2i}\sim t\left(0,\sigma_{e2}^{2},\nu \right)$), where ν is the degree of freedom for the Student's *t* distribution. Although the two models M1 and M2 have the same structure, they are different distributional models. Similarly, when the treatment is categorical, there are also two types of models: the cat-normal-based model (M3) where $e_{i}\sim N\left(0,\sigma^{2}\right)$, and the cat-robust model (M4) where $e_{i}\sim t\left(0,\sigma^{2},\nu \right)$, and ν is the degree of freedom for the Student's *t* distribution.

The two-stage causal modeling can also handle missing data which are almost inevitable in practice. Model estimation with missing data can be conducted relatively easily in the Bayesian framework. When the missingness is ignorable (i.e., missing completely at random or missing at random), missing values are handled using multiple imputation techniques. When the missingness is non-ignorable (missing not at random), a selection model is applied. A probit link function is added to the second stage of the model so that the missingness in the outcome variable is explained by the link function. For continuous treatment variables, selection models can be added to the traditional two-stage model and the robust model using *t* distributions, denoted as cont-normal-selection model (M5) and con-robust-selection model (M6), respectively. For categorical treatment variables, two added-on selection models are also proposed, denoted as cat-normal-selection model (M7), and cat-robust-selection model (M8). Table 1 presents an overview of the model types and the corresponding data types that each model is suited for.

**Table 1:** Taxonomy of models.

**Types of the model**	**Types of outcome data**				
	**Normally distributed**	**Complete**	**Having outliers**	**Ignorable missing**	**Non-ignorable missing**
Cont-normal model	YES	YES	NO	YES	NO
Cont-robust model	YES	YES	YES	YES	NO
Cont-normal-selection model	YES	YES	NO	YES	YES
Cont-robust-selection model	YES	YES	YES	YES	YES
Cat-normal model	YES	YES	NO	YES	NO
Cat-robust model	YES	YES	YES	YES	NO
Cat-normal-selection model	YES	YES	NO	YES	YES
Cat-robust-selection model	YES	YES	YES	YES	YES

## 2. Overview of the ALMOND Package

The ALMOND package has been developed and can be used to estimate the eight models (M1-M8) described above. Each model can be directly applied with a function in the ALMOND package. The package uses the R2OpenBUGS package in R to call the open-source Bayesian software OpenBUGS (Thomas et al., 2006) to conduct the Bayesian analyses. End-users need to have both R and OpenBUGS installed for the analysis. Results mimic what are reported from OpenBUGS. This section presents an overview of the package and introduces the markov chain Monte Carlo (MCMC) algorithm used in the package. This section illustrates the application of the ALMOND package, using two empirical examples from the existing literature. The ALMOND package can be installed in R using the *devtools* package (Wickham and Chang, 2016) since the source code is available on GitHub. Thus, the first step is to load the *devtools* package, and then install the ALMOND package using the *install_github()* function:

library(devtools)
devtools::install_github('dingjshi/ALMOND')


### 2.1. Components of the Package

Each of the eight models mentioned in the previous section has a corresponding function to represent. Specifically, for continuous treatment variables, Models M1, M2, M5, and M6, representing the cont-normal-based model, the cont-robust model, the cont-normal-selection model and the cont-robust-selection model, respectively, can be programmed using the functions *ts.nnormal, ts.nrobust, ts.nnormal.s* and *ts.nrobust.s*; similarly, for categorical treatment variables, Models M3, M4, M7, and M8 have the corresponding functions *gts.nnormal, gts.nrobust, gts.nnormal.s* and *gts.nrobust.s* to represent the cat-normal-based model, the cat-robust model, the cat-normal-selection model and the cat-robust-selection model, respectively.

Each of the model functions has multiple arguments, and only the first two arguments are required for the non-selection models. An additional missingness indicator argument is needed if an added-on selection model is applied. Details about the Bayesian methods can be flexibly tailored in other optional arguments of the model functions. An argument breakdown is illustrated below.

1. Required arguments.
  - The formula of the model. The model formula comprises three parts: the outcome variable, the independent variables, and the InsVs. In the formula, a tilde (~) and a vertical slash (|) are used to connect the three parts:
    $$\begin{array}{l} outcome\;\sim \;independent\;variables|InsVs. \end{array}$$
    The formula shows that the outcome is modeled from two sources of variations: the independent variables to the left of the vertical slash and the InsVs to the right of the vertical slash. The numbers of independent variables and InsVs are at least one for each and theoretically, have no upper limits. The outcome can only contain one variable. Multiple variables can be added as independent variables and InsVs, connected by the plus (+) sign. In the independent variables part, the causal treatment variable is always the first element and any variables added after the first element are covariates at the second stage of the causal model. The package does not currently have the flexibility to accommodate multiple causal treatment variables or the multivariate causal analysis. In the InsVs part, all elements are treated as the InsVs from the first stage, all together predicting the estimated treatment effect. For a detailed explanation of the method, see Shi and Tong (accepted).
  - The data. Datasets must be in the form of a data frame.
  - The missingness indicator (for added-on selection models only). The *m.ind* argument is used to identify the missingness indicator variable in the new data and is only required when the added-on selection model is used. For other models, this argument is not needed. When the added-on selection model is applied, users need to create a new variable and add it to the original data to indicate the missingness status of the outcome variable. The missingness indicator is a binary variable. It equals 1 if the outcome value is missing, and 0 otherwise.
2. Optional arguments.
  - The ALMOND package has the flexibility to allow users to specify any key elements of their interest in Bayesian statistics, including priors distributions, starting values, number of Markov chains, length of burn-in periods, etc. Other optional arguments include reporting the Deviance Information Criterion (DIC) and technique details of the error message.
  - Prior distributions. The prior arguments specify the prior distributions and include *b0* and *B0, g0* and *G0*, u0 and U0 and e0 and E0. The priors for the regression coefficients at the first and second stages of the model are normal distributions with b0, B0 and g0, G0 representing the location and precision hyperparameters, respectively. The priors for the error variances at the first and second stages of the model are inverse gamma distributions with u0, U0, and e0, E0 representing shape and scale hyperparameters, respectively. The four hyperparameters for the priors of the regression coefficients (b0, B0, g0, and G0) can be specified as vectors (if there are multiple predictors at the two stages and priors for the regression coefficients are designed to be different), or one numerical value (if priors for all the regression coefficients are designed to be the same). For example, for a model with one treatment (X1), two additional covariates (X2-X3) and two InsVs (Z1-Z2), the prior argument should be specified using the logic such as: b0 = c(0,0.5), B0 = c(1000,10000), g0 = c(0, 0.4, 0.7), G0 = c(100,1000,10000).
  - The basic model uses non-informative priors for all model parameters. If no prior is specified in the function, the model will be estimated with non-informative priors as default. We encourage users to use informative priors or the data dependent priors (i.e., using the maximum likelihood estimates as the hyperparameters) to obtain more reliable parameter estimates (McNeish, 2016; Shi and Tong, 2017a). The default priors in the package are data-dependent priors. In addition, the numerical values of the hyperparameters can be specified using the prior arguments. If users want to use other prior distributions rather than normal or inverse gamma distributions, see the advanced feature in the argument that is explained in the public housing voucher program example (Example 2) of the article.
  - Starting values. The default starting values for the parameters with the Bayesian algorithm is the frequentist estimates from conventional two-stage-least-squares models (Angrist and Imbens, 1995). Users are encouraged to specify other reasonable or multiple starting values (accompanied by multiple chains) of their choice.
  - Other details for Bayesian methods. Users have the flexibility to specify the number of Markov chains, the total number of iterations per chain, the length of the burn-in period and the thinning rate. The default number of Markov chains is one. The default total iterations per chain are 10,000 for non-selection models, and 50,000 for added-on selection models. The default length of burn-in (i.e., number of iterations to discard at the beginning) is half of the number of stated iterations, meaning to discard the first half of iterations, and the default thinning rate is 1.
  - Bayesian model fit index. DIC is used as the fit index in the two-stage Bayesian causal modeling and is reported as default.
  - Model convergence. The output lays the groundwork for reporting Bayesian model convergence. For example, when more than one chain is used, the potential scale reduction factor (Gelman and Rubin, 1992) can be requested by specifying *output$summary[,”Rhat”]*. Other model convergence measures such as the Geweke statistics (Geweke, 1992) can be requested using the coda package (Plummer et al., 2006), which has been imported in ALMOND.
  - Model debugging phase. By default, *debug=FALSE* in the function means not to report the technical details. The *debug=TRUE* is recommended to be stated when an error message appears, so users can use the information to debug their codes.
3. A special feature of the package.
  A unique advantage of the ALMOND package is that it allows users to either directly apply the models from the package or specify their own Bayesian models from scratch. It is not uncommon that users may want to assign prior distributions of their own preference rather than using the default distributions; users may also be interested in applying other robust models not specified in the package [e.g., fitting a robust model at the first stage (e.g., Shi and Tong, 2017b)]. The advanced feature of the ALMOND package has been designed especially for these types of scenarios, in that users can build their Bayesian models from scratch. In particular, the *advanced* argument together with the *adv.model* argument is designed for advanced users to self-define their model of interest. Specifically, the *advanced* argument serves as a switch to the advanced feature, when *advanced=TRUE* is specified, the switch is on and users can write their own Bayesian model and pass it to the *adv.model* argument. The public housing voucher program example (Example 2) illustrates the application of the advanced feature.
  This special feature of the package accommodates the need for a wide range of Bayesian users, from those who are more advanced to those who are less familiar with Bayesian methods and programming. On one hand, the ALMOND package allows users to write their own Bayesian functions and distributions, like popular Bayesian software programs such as BUGS (Spiegelhalter et al., 2003) and JAGS (Plummer et al., 2003). This could be a merit for advanced Bayesian users. On the other hand, users who are less familiar with Bayesian programming may find it difficult to take advantage of the above-mentioned Bayesian software and apply Bayesian methods. The ALMOND package conveniently fits the Bayesian robust modeling framework for them and handles the outliers and missing values in the empirical data. A detailed example of both approaches is illustrated in Example 2.

### 2.2. Gibbs Sampling Algorithm for the Computation

In the ALMOND package, the Gibbs sampling algorithm is used to obtain the parameter estimates for the two-stage causal model with InsVs. In particular, data augmentation methods are implemented. The estimation steps of the Gibbs sampling algorithm with data augmentation for the cont-robust model with missing data are given below. Gibbs sampling algorithms for other models are similar. Shi and Tong (accepted) provided a detailed derivation of the posterior distributions.

1. Start with initial values $\pi_{1}^{\left(0\right)}$, $\pi_{2}^{\left(0\right)}$, $\sigma_{e1}^{2\left(0\right)}$, $\sigma_{e2}^{2\left(0\right)}$, ν^(0)^, $\omega_{i}^{\left(0\right)}$, $y_{mis}^{\left(0\right)}$ where $\pi_{1}^{\left(0\right)}={\left(\pi_{10}^{\left(0\right)},\pi_{11}^{\left(0\right)}\right)}^{\prime }$ and $\pi_{2}^{\left(0\right)}={\left(\pi_{20}^{\left(0\right)},\pi_{21}^{\left(0\right)}\right)}^{\prime }$.
2. Assume at the *jth* iteration, we have $\pi_{1}^{\left(j\right)}$, $\pi_{2}^{\left(j\right)}$, $\sigma_{e1}^{2\left(j\right)}$, $\sigma_{e2}^{2\left(j\right)}$, ν^(*j*)^, $\omega_{i}^{\left(j\right)}$, $y_{i,mis}^{\left(j\right)}$, where $\pi_{1}^{\left(j\right)}={\left(\pi_{10}^{\left(j\right)},\pi_{11}^{\left(j\right)}\right)}^{\prime }$ and $\pi_{2}^{\left(j\right)}={\left(\pi_{20}^{\left(j\right)},\pi_{21}^{\left(j\right)}\right)}^{\prime }$.
3. At the (*j* + 1)*th* iteration,
  3.1. Sample $\pi_{1}^{\left(j+1\right)}$ from $p\left(\pi_{1}|\sigma_{e1}^{2\left(j\right)},{\text{x}}_{i},{\text{Z}}_{i},i=1,\ldots ,N\right)$;
  3.2. Sample $\sigma_{e1}^{2\left(j+1\right)}$ from $p\left(\sigma_{e1}^{2}|\pi_{1}^{\left(j+1\right)},{\text{x}}_{i},{\text{Z}}_{i},i=1,\ldots ,N\right)$;
  3.3. Sample ${\text{y}}_{i,mis}^{\left(j+1\right)}$ from $p({\text{y}}_{i,mis},|\pi_{2}^{\left(j\right)},\sigma_{e2}^{2\left(j\right)},{\hat{\text{x}}}_{i},{\text{y}}_{i,obs},$ *i* = 1, …, *N*);
  3.4. Sample $\sigma_{e2}^{2\left(j+1\right)}$ from $p(\sigma_{e2}^{2}|\pi_{2}^{\left(j\right)},{\hat{\text{x}}}_{i},\omega_{i}^{\left(j\right)},{\text{y}}_{i,obs},{\text{y}}_{i,mis}^{\left(j\right)},$ *i* = 1, …, *N*);
  3.5. Sample ν^(*j*+1)^ from $p\left(\nu |\omega_{i}^{\left(j\right)},i=1,\ldots ,N\right)$;
  3.6. Sample $\omega_{i}^{\left(j+1\right)}$ from $p(\omega_{i}|\nu^{\left(j+1\right)},\sigma_{e2}^{2\left(j+1\right)},\pi_{2}^{\left(j\right)},{\hat{x}}_{i},{\text{y}}_{i,mis}^{\left(j+1\right)},{\text{y}}_{i,obs},$ *i* = 1, …, *N*);
  3.7. Sample $\pi_{2}^{\left(j+1\right)}$ from p(π2|ωi(j+1),σe22(j+1),x^i,yi,mis(j+1),yi,obs, *i* = 1, …, *N*).
4. Repeat Step 3.

## 3. Applications of the ALMOND Package

### 3.1. Example 1–Early Childhood Reading Achievement

The first example is motivated by a study on the effect of children's relative age on early childhood reading achievement. Zhong and Hoxby (2012) used the conventional frequentist two-stage least squares (2SLS) model approach to examine the relative age effect, using the Early Childhood Longitudinal Study—Kindergarten Cohort (ECLS-K) data. In Zhong and Hoxby (2012)'s original work, pairwise deletion was used to handle missing data and the study assumed that the outcome data were normally distributed. Answering the same research question, Shi and Tong (accepted) applied a proposed Bayesian robust two-stage causal modeling technique to deal with missing data and potential outliers in the ECLS-K data. This example provides a tutorial to the ALMOND package for implementing the Bayesian robust two-stage causal modeling techniques. A subset of ECLS-K data is built in the package.

ECLS-K is a large, nationally representative survey that tracks children from kindergarten through the eighth grade. A subset of the ECLS-K data is available in the package and can be read in as *data(subECLSK)*. The dataset contains missing values and few outliers. To replicate previous studies, all variables of interest are kept the same as in the original studies. Specifically, the outcome variable of the study is the reading scores from the third grade. The treatment variable/predictor is children's relative age (i.e., the difference between a child's individual age and the median age of all children in the same grade) measured in months. Because relative age could be endogenous to parents' observations of their children (Zhong and Hoxby, 2012), and merely using children's relative age to predict the reading outcome as in conventional linear models may lead to selection bias, because of potentially omitted confounding factors (e.g., one's intellectual maturity which may affect the outcome—academic achievement but is likely to correlate with the treatment—one's relative age). One solution is to incorporate an InsV which is correlated with the causal treatment but has no direct effect on the outcome variable so that the confounding effects are extracted by the InsVs. Authors of the original study found a positive effect from kindergarten through grade five in reading (Zhong and Hoxby, 2012), and that an ideal InsV could be the predicted relative entrance age (interaction between a child's birthday and the state-level cut-off date for the school enrollment), as this variable is highly correlated with the actual relative age, but uncorrelated with the causal outcome itself or other unobserved determinants of academic achievements such as maturity or aptitude (Allhusen et al., 2007). With the inclusion of the predicted relative entrance age as the InsV, the local average treatment effect (LATE) is the causal effect of the early childhood relative age on reading achievement for children who enroll in the same year that meets the state-level requirements.

The example dataset has a continuous treatment variable and contains 600 participants. The missingness rate in the outcome reading score is 20%. All other variables have complete data. Few outliers are in the outcome variable. The cont-normal-selection model (M5) is appropriate to apply here and the *ts.nnormal.s()* function in the ALMOND package is used. Following Zhong and Hoxby (2012), the demographic information including gender, race, socio-economic status, the number of siblings, and parental education levels are included in the analysis as covariates to control other potential confounding effects. Because demographic variables are covariates exclusively for the causal relations, but not for the relations between the InsVs and the treatment variables, the demographic covariates are included only to the second stage.

The added-on selection model is used in this example. The outcome variable is the reading scores and the corresponding missingness indicator variable for the reading outcome data, *mis.ind.read* is stated using the *m.ind* argument, as *m.ind=subECLSK$mis.ind.read*. Because the added-on selection model is relatively complicated as it handles the non-ignorable missingness in the outcome variable, a longer Markov chain is usually preferred. The default burnin length is 50,000 for the added-on selection model. In this example, a Markov chain of 100,000 with the first 60,000 iterations as the burn-in period is requested. The code for this example is illustrated below. The data-dependent priors (DDP) are used as the default priors in the analysis. Specifically, parameter estimates of the coefficients at both stages and the associated standard errors (SEs) are first estimated by the traditional frequentist two-stage ordinary least squares model (2SLS, Angrist and Imbens, 1995). These estimates and the associated SEs are then used as the hyperparameters in the prior specifications of the model. In this way, prior specifications in the Bayesian analysis are more informative.


output = ts.nnormal.s(readingIRT~relAge+
gender+race+ses+numsib+parentedu|PredEnt,
data=sub ECLSK, m.ind = subECLSK$mis.ind.
read, n.burnin = 60000, n.iter=100000)


**Figure 1:**
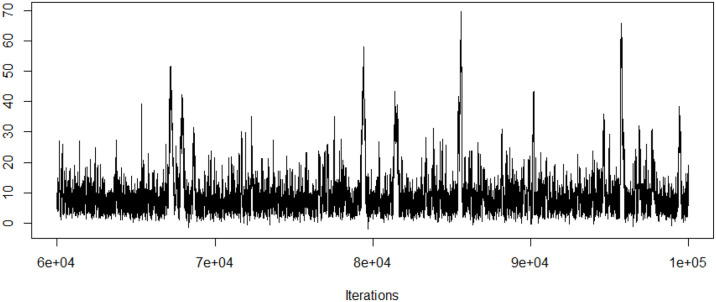
Traceplot of the LATE estimate.

Geweke diagnostic is used to assess the model convergence. Geweke z-score is 1.18 and shows convergence of the Markov chains. Traceplot of the LATE estimate is also presented in Figure 1. Both geweke z statistic and traceplot are requested from the coda package with corresponding codes illustrated below.


traceplot(as.mcmc.list(output)[[1]][,5])
geweke.diag(as.mcmc.list(output)[[1]][,5])


#### 3.1.1. Results of the Reading Ability Study

Parameter estimates, their standard errors and 95% credible intervals of the cont-normal-selection model are reported in Table 2. The s2.slope parameter is also the LATE, often the parameter of interest in a study. The LATE estimate means that for a one unit increase in the relative age of kids who meet the predicted entrance age requirement, their reading score is expected to increase 9.5 points, holding other variables constant. The average reading score for all kids who meet the predicted entrance age requirement is 105.7 points, based on the intercept estimate at stage 2. The intercept and slope estimates at the first stage reflect the relation between the InsVs (the predicted relative entrance age) and the causal treatment (the relative age of school children). In other words, for a child with a one unit increase in the predicted relative entrance age, the relative age is 0.116 higher. The number is significant but not large in quantitaty, as the predictive relative entrance age is closely correlated with the relative age. The remaining estimates are the coefficient estimates of the covariates and the error variance estimates at both stages.

**Table 2:** Parameter estimates for early childhood reading achievement.

	**Est**.	**S.E**.	**CI.L**	**CI.U**
stage1.intercept	−0.100	0.300	−0.600	0.500
stage1.slope	0.116	0.038	0.047	0.193
stage2.intercept	105.7	6.600	91.100	117.100
LATE	9.500	7.400	2.600	31.400
gender.coefficient	4.503	1.702	1.203	7.900
race.coefficient	−1.070	0.473	−1.998	−0.141
ses.coefficient	−1.421	0.739	−2.874	0.027
nsib.coefficient	−1.341	0.858	−3.020	0.348
parentEdu.coefficient	15.260	2.155	11.020	19.490
stage1.variance1	0.087	0.005	0.077	0.097
stage2.variance2	0.003	1.91E-04	0.003	0.003

*1. Est., Estimate; S.E., Standard Error; CI.L, 95% Lower credible interval; CI.U, 95% Upper credible interval*.

*2. DIC for this model is 7936*.

### 3.2. Example 2–Public Housing Voucher Program

This example investigates the effect that receiving and living in a larger housing unit as part of a public housing voucher program has on improving participants' housing qualities. The participation status of the voucher program is a dichotomous data and a generalized Bayesian two-stage causal model is used in this example. A similar research question has been examined by Currie and Yelowitz (2000). In Currie and Yelowitz (2000)'s studies, potential selection bias occurs as families participating in the voucher programs trade physical housing amenities for rental payment reductions which results in negative effects for the housing qualities, and an InsV is necessary. Because previous theories have supported that a household having the sex decomposition of an extra kid (i.e., having at least one girl and one boy) is entitled to a larger housing unit as they often don't share bedrooms, this example chooses whether there is an extra sex decomposition kid in the household as an instrument (1 if yes and 0 otherwise). The study investigates the partial treatment effect of the public housing voucher program on participants who have one girl and one boy (i.e., having sex decomposition) in the household. The outcome variable is the housing quality measured as residents' housing ratings. The causal treatment of interest is the participation status in the public housing voucher program (1 if participated and 0 otherwise).

**Figure 2:**
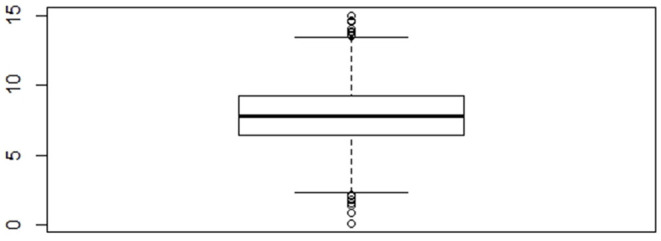
Boxplot of the housing quality.

Data are simulated based on the descriptive statistics in Currie and Yelowitz (2000)'s study. The simulated data can be read in as *data(simVoucher)* in the ALMOND package. For illustration purposes, gender and marital status of the household head are used as covariates in this example. The boxplot of the outcome housing quality (see Figure 2) shows evidence of extreme values and extreme values could be potential outliers. A generalized cat-robust model (M4) is fitted to the data and the *gts.nrobust()* function from the ALMOND package is used. The model can be specified using either the basic built-in feature as described in Example 1, or the advanced user-defined feature which allows more flexibility to users. This example illustrates the use of the two features, with an emphasis on the advanced feature.

#### 3.2.1. The Basic Built-In Feature

The application of the basic built-in feature follows a similar logic to what was described in Example 1. Users only need to place variables in the corresponding formula argument and read in the data, and the model functions will take care of the rest model specifications. Other arguments can be left as optional. Under certain specifications, results from the basic and advanced features should be the same. The code for the basic feature is provided below. In the example code, the rating of the housing qualities is the outcome variable and is placed on the left side of the tilde (~) symbol in the model specification argument; the treatment and other covariates are placed on the right of the tilde (~) symbol and to the left of the verticle (|) symbol. The variable listed to the right of the verticle (|) symbol is the InsV, which is whether there is an extra bedroom in the participated household. Note that there could be multiple InsVs in a study, which could be reflected in the model specification argument, with all InsVs connected by the addition (+) symbol on the right of the verticle (|). In this example, there is one InsV.


output=gts.nrobust(hmRating~voucher
+headFemale+headMarried|extraBed,
data=simVoucher)


The output serves as the basis for further Bayesian analysis, such as making visualizations, conducting MCMC diagnostics or computing information for posterior distributions. Readers can save the output as the MCMC object using the function *output.mcmc = as.mcmc.list(output)* and implement further Bayesian analysis as necessary. Note that a generalized causal model can be applied only when the treatment variable is binary with the basic built-in feature. An error message will appear if the categorical treatment variable has more than two levels. If users wish to analyze categorical but not binary data, they can consider using the advanced user-defined feature as illustrated below.

#### 3.2.2. The Advanced User-Defined Feature

The basic feature limits the treatment data to be continuous or binary, as well as the priors of the parameters to follow certain types of distributions (e.g., normal or inverse gamma). The advanced feature gives users the flexibility to specify any model (e.g., a multinomial logistic model in the presence of the nominal outcome) and any prior distributions. Users can self-define the model part using the OpenBUGS language (Lunn et al., 2009). The other parts (e.g., starting values, number of chains) in the function remain the same as those in the basic feature. Specifically, the *advanced=TRUE* and *adv.model* arguments serve as the “twin” arguments to turn on the advanced user-defined feature. The *advanced=TRUE* argument informs the program that the user will self-define its own model, and *adv.model* argument calls the name of the function that the user previously defined. There will be three parts in the self-defined *adv.model* argument. A breakdown of each part in the argument is illustrated in details as below.

Three parts consist of the self-defined *adv.model* argument – the model part, the prior part and the parameter reformatting part. The following example uses the advanced feature to re-do the same analysis illustrated from the basic feature.


my.model<- function(){
 for (i in 1:N){
   logit(p[i]) <- beta0 + beta1*z[i]
   x[i]~dbern(p[i])
   muY[i] <- gamma0 + gamma1*p[i] + gamma2
   *x1[i] + gamma3*x2[i]
  y[i]~dt(muY[i], pre.u2, df)
}
   beta0~dnorm(0,1E-6)
  beta1~dnorm(0,1E-6)
  gamma0~dnorm(0,1E-6)
  gamma1~dnorm(0,1E-6)
  gamma2~dnorm(0,1E-6)
  gamma3~dnorm(0,1E-6)

   pre.u2~dgamma(.001,.001)

   df~dunif(0,100)

   s1.intercept <- beta0
 s1.slope1 <- beta1
 s2.intercept <- gamma0
 s2.slope1 <- gamma1
 s2.slope2 <- gamma2
 s2.slope3 <- gamma3 
 df.est <- df
 var.e.s2 <- 1/pre.u2
}


First, users define their own model function and assign it a name (e.g., my.model). Going into the function, users specify the model and use the tilde (~) symbol to represent the distribution of the data. The example uses a logia function to model the binary treatment variable and assumes a Student's *t* distribution for the outcome variable as part of the robust procedure. The data distributions are described with corresponding hyperparameters as below.


x~dbern (p)
y~dt (muY, pre.u2, df)


Next, prior distributions are defined for model parameters. In the example, non-informative priors are specified for each parameter, where regression coefficients from both stages follow normal distributions with large variances, and error variance parameters follow the inverse gamma distribution. Note that there is an additional parameter estimate in the cat-robust model (M4) - the degrees of freedom of the Student's *t* distribution. The degrees of freedom can be fixed a priori or estimated from the model. Users have the flexibility to either assign an informative prior to the function argument or specify the advanced model of their own. Finally, in the last part users reformat the names of the parameters that will appear in final results.

This self-defined model is used as an advanced feature in the original *gts.nrobust()* function. The remaining specifications are the same as those in the basic feature.


gts.nrobust(hmRating~voucher+headFemale+
headMarried|extraBed,
data=simVoucher, advanced=TRUE, adv. model=
my.model)


Both illustrations for the basic and advanced features in Example 2 are doing the same analysis. Note that although flat priors (e.g., beta0 ~ dnorm(0,1E-6)) are used in the example code, authors always have the flexibility to specify a non-informative prior with less variance [e.g., beta0 ~ dnorm(0,1E-2)]. One big advantage of the ALMOND package is that users can flexibily specify a flat prior or a non-informative prior either from the advanced feature (as in the illustrated code above) or even in the basic feature. For example, if users desire to specify a non-informative prior for the LATE parameter (e.g., gamma1) from the basic feature, the corresponding code would be as below, where the argument G0 represents a vector of variance hyperparameters for the priors for the second-stage coefficients.


output=gts.nrobust (hmRating~voucher+
headFemale+headMarried|extraBed,G0=c(1e-06,
1e-02,1e-06,1e-06),data=simVoucher)


The parameter estimates, their standard errors and 95% credible intervals of the cat-robust model are presented in Table 3. From the LATE parameter estimate, it was found that the voucher program participants who have the sex decomposition (i.e., at least one girl and one boy) in the household are more likely to have better housing qualities than the non-participants who also have the sex decomposition in the household. The second-stage intercept estimate shows that program non-participants who have the sex decompostition in the household have a baseline unit score of 6.6 for the housing quality. Among households that have the sex decomposition, program participants have 1.20 unit higher housing quality score than the non-participants. Covariates of gender and marital status do not have significant effects of affecting program participation on the housing qualities.

**Table 3:** Parameter estimates for the voucher program effect.

	**Est**.	**S.E**.	**CI.L**	**CI.U**
stage1.intercept	1.80	0.10	1.70	1.90
stage1.slope	2.00	0.20	1.60	2.40
stage2.intercept	6.60	0.20	6.20	7.00
LATE	1.20	0.20	0.80	1.60
gender.coefficient	–0.10	0.10	–0.30	0.10
marital status.coefficient	0.10	0.10	0.00	0.30
stage2.variance	4.20	0.10	3.90	4.50
df.est	34.50	7.90	17.80	46.70

*1. Est., Estimate; S.E., Standard Error; CI.L, 95% Lower credible interval; CI.U, 95% Upper credible interval*.

*2. DIC for this model is 9497*.

## 4. Summary

Selection bias is a common and practical challenge in observational studies and quasi-experimental designs for causal inference. A Bayesian robust two-stage causal modeling technique was proposed to address the issue of selection, which simultaneously accommodates the non-normality of the data, handles ignorable and non-ignorable missing data, and provides correct SE estimates for the partial treatment effect estimation, while still performs well under ideal situations (i.e., data are normally distributed and completely observed) (Shi and Tong, accepted). The ALMOND package in R is developed to directly apply this Bayesian robust two-stage causal modeling approach. Previous causal studies have focused mostly on categorical treatment variables, the ALMOND package can be used for both categorical and continuous treatment variables.

The ALMOND package has several distinct advantages. First, the package adopts a Bayesian perspective to mitigate selection bias. Most current causal inference software uses a frequentist approach, which hardly handles non-normal data or missing data, and has the potential problem of obtaining unreliable standard error estimates. These issues can be simultaneously addressed in one model using the Bayesian modeling framework in the ALMOND package. Second, the package has a flexible option for users to either directly apply Bayesian robust two-stage causal models or write their own Bayesian models from scratch. Therefore a broad range of audiences with various statistical backgrounds and programming knowledge can benefit from using the package. The ALMOND package fills the methodology gap by adding the Bayesian approach to existing software. The package is beneficial and accessible to a broad range of methodologists and applied researchers in social and psychological studies, which eventually help advance the field of psychology and statistics. For this pursuit, here we provide a tutorial for the newly developed R package to facilitate the comprehension and application of the Bayesian robust two-stage causal model with InsVs. Two examples using simulated data based on existing empirical research literature are used as illustrations of applying the models using the ALMOND package.

## Data Availability Statement

Publicly available datasets were analyzed in this study. This data can be found here: https://nces.ed.gov/ecls/kindergarten.asp.

## Author Contributions

DS designed the study, drafted the manuscript, analyzed the data, and created the R package. XT designed the study, contributed to the analysis of the data, to the writing of the manuscript, and to the development of the R package. MM contributed to the design of the study, to the analysis of the data, to the writing of the manuscript and to the development of the R package.

### Conflict of Interest

The authors declare that the research was conducted in the absence of any commercial or financial relationships that could be construed as a potential conflict of interest.
