# Corrigendum: A Bayesian Approach to the Analysis of Local Average Treatment Effect for Missing and Non-normal Data in Causal Modeling: A Tutorial With the ALMOND Package in R

**DOI:** 10.3389/fpsyg.2020.02156

**Published:** 2020-09-04

**Authors:** Dingjing Shi, Xin Tong, M. Joseph Meyer

**Affiliations:** ^1^Department of Psychology, University of Oklahoma, Norman, OK, United States; ^2^Department of Psychology, University of Virginia, Charlottesville, VA, United States

**Keywords:** instrumental variables (IV), bayesian method, robust method, missing data, selection bias, R package, causal modeling, local average treatment effect

In the published article, there was an error regarding the affiliations for Dingjing Shi. As well as having affiliation University of Oklahoma, they should also have University of Virginia.

The authors apologize for this error and state that this does not change the scientific conclusions of the article in any way. The original article has been updated.

